# Plantar Fasciitis as a Potential Early Indicator of Elevated Cardiovascular Disease Risk

**DOI:** 10.7759/cureus.62007

**Published:** 2024-06-09

**Authors:** Kossay Elabd, Loay Basudan, Mohammad A Alomari, Ayman Almairi

**Affiliations:** 1 Family Medicine, King Faisal Specialist Hospital and Research Centre, Riyadh, SAU; 2 Medicine, Yarmouk University, Irbid, JOR; 3 Medicine, Alfaisal University College of Medicine, Riyadh, SAU

**Keywords:** dyslipidemia, hypertension, diabetes, risk factors, chronic heel pain, systemic inflammation, cardiovascular disease, c-reactive protein, plantar fasciitis

## Abstract

Background

Plantar fasciitis is characterized by heel pain and is often associated with extended periods of walking or standing, improper footwear, and biomechanical imbalances. This condition primarily affects the bottom of the foot, particularly the area where the heel meets the arch. Despite its prevalence, the potential systemic effects, especially the relationship with cardiovascular disease (CVD) risk factors, require further illumination. This study explores the association between chronic plantar fasciitis and elevated C-reactive protein (CRP) levels in individuals with cardiovascular risk factors.

Methods

A cross-sectional study of 400 patients with foot or ankle pain was initially assessed clinically and with ultrasound or MRI scans. After excluding those with confounding factors for elevated CRP, 295 patients with concurrent diabetes, hypertension, or dyslipidemia were analyzed. We investigated the correlation between plantar fasciitis and elevated CRP levels, defined as >1 mg/L, in the context of cardiovascular risk assessment.

Results

The study indicated that nearly half of the patients suffering from foot or ankle pain were diagnosed with plantar fasciitis, accounting for 47.8% of cases. A statistically significant association was observed between plantar fasciitis and elevated CRP levels (p=0.035). Furthermore, a substantial correlation was found between high BMI and plantar fasciitis, but no gender-specific disparity was noted. Elevated CRP levels were significantly associated with diabetes, hypertension, and dyslipidemia.

Discussion

A definitive cause-and-effect relationship between plantar fasciitis and systemic inflammation has not been established; our study suggests that chronic plantar fasciitis may be more than a localized condition and could be indicative of systemic inflammation, which is known to be a factor in atherosclerosis and CVD. The observed correlation between increased CRP levels and plantar fasciitis suggests that plantar fasciitis might be a clinical indicator of systemic inflammation and could improve the assessment of CVD risk.

Conclusions

Elevated levels of CRP, associated with chronic plantar fasciitis, suggest a link to systemic inflammation, which could elevate the risk of CVD. Identifying plantar fasciitis as a marker for systemic inflammation in patients with CVD risk factors, including diabetes, hypertension, and dyslipidemia, underscores the importance of thorough cardiovascular evaluations in individuals with persistent heel pain. Further longitudinal and interventional research is essential to substantiate these preliminary findings and understand their impact on CVD risk management and treatment.

## Introduction

C-reactive protein (CRP) is a protein the liver produces in response to inflammation. An elevated CRP level is a marker indicating inflammation in the body, but it does not specify the cause or location of the inflammation. Several conditions and situations can increase CRP levels, including infections, chronic inflammation such as rheumatoid arthritis, inflammatory bowel diseases, autoimmune diseases, and chronic obstructive airway diseases. In addition, cancer, physical trauma or surgery, smoking, obesity, diabetes, and cardiovascular diseases can increase CRP levels [1-5].

CRP and high-sensitivity C-reactive protein (hs-CRP) are both markers used to detect inflammation in the body. However, they differ mainly in their sensitivity and the specific clinical contexts in which they are used. Traditional CRP tests measure protein levels that indicate acute inflammation, such as infection or injury. The test can detect CRP levels that are significantly elevated, usually above 10 mg/L. In contrast, high-sensitivity CRP tests are designed to detect lower levels of CRP and can measure concentrations as low as 0.3 to 10 mg/L. This enhanced sensitivity allows hs-CRP to assess the risk of chronic conditions, particularly cardiovascular diseases, where inflammation plays a subtle yet significant role in atherosclerosis development [6-8]. The CRP levels are categorized into three categories based on ASCVD risk: low risk: <1 mg/L, average risk: 1-3 mg/L, and high risk: >3 mg/L [6-9].

There is a well-documented link between CRP, particularly hs-CRP, and the risk of atherosclerotic cardiovascular disease (ASCVD), with several studies validating their positive association. Elevated hs-CRP levels specifically have been linked to a greater risk of ASCVD [7,8].

While high CRP levels are considered alongside other factors for cardiovascular risk assessment, they are not the sole diagnostic markers. Instead, they are part of a broader evaluation, providing additional insight when other risk indicators are ambiguous. High CRP levels may indicate heightened arterial inflammation, contributing to atherosclerosis progression and an elevated risk of ischemic events.

Plantar fasciitis is commonly known for its heel pain due to plantar fascia inflammation. It is believed to result from repetitive stress and mechanical strain on the plantar fascia. It is often related to extensive walking or running, unsuitable footwear, being overweight, or foot mechanics issues like high arches or flat feet. The condition is characterized by sharp pain near the heel, especially pronounced with initial steps after resting. Diagnosis is typically clinical, based on patient history and physical examination, and sometimes involves radiological imaging to exclude other causes of heel pain [10-13].

Contributors to the development of plantar fasciitis encompass biomechanical disparities, excessive body weight, the aging process, intense physical activities, degenerative tissue changes, and unsuitable footwear. Although a direct causal link between plantar fasciitis and metabolic conditions such as diabetes, hypertension, or dyslipidemia has not been firmly established, there is an observed higher occurrence of plantar fasciitis in individuals with these health issues, potentially due to the common factor of obesity [11,12,14,15].

This research intends to examine the potential relationship between chronic plantar fasciitis and an increased risk of cardiovascular diseases in individuals with other cardiovascular risk factors, like diabetes, hypertension, and dyslipidemia. We will investigate whether chronic heel pain caused by plantar fasciitis correlates with elevated CRP levels, reflecting systemic inflammation. The direct connection between plantar fasciitis and increased CRP levels has not been conclusively established in existing literature [15].

## Materials and methods

This non-interventional, cross-sectional study was conducted at the Family Medicine outpatient clinics from January 1, 2021, to January 1, 2023, aiming to investigate the association between plantar fasciitis and elevated CRP levels in individuals with cardiovascular risk factors. A total of 400 patients presenting with foot or ankle pain were initially assessed, and after applying inclusion and exclusion criteria, 295 patients were included in the final analysis. The inclusion criteria encompassed patients aged 18 years or older, presenting with foot or ankle pain, and having at least one cardiovascular risk factor, such as diabetes mellitus, hypertension, or dyslipidemia. Exclusion criteria ruled out patients with acute or chronic inflammatory diseases like rheumatoid arthritis, gout, or Crohn's disease, autoimmune disorders, any cancer, recent trauma, surgical procedures, current smokers, and those with infections or conditions elevating CRP levels beyond cardiovascular risk factors.

Participants were divided into two groups based on the etiology of their foot or ankle pain: those with plantar fasciitis and those with other causes, such as flatfoot or peripheral neuropathy. The diagnosis was confirmed clinically and through imaging techniques, including ultrasound and MRI scans. Data collected included demographic information, medical history, laboratory results, and CRP levels, measured using a high-sensitivity assay and considered elevated if greater than 1 mg/L. The prevalence of diabetes, obesity, hypertension, and dyslipidemia was compared between the two groups, and the association between plantar fasciitis and elevated CRP levels was analyzed using the chi-square test. Descriptive statistics for continuous variables were reported as mean with standard deviation (SD) and categorical variables as frequencies and percentages, with the level of statistical significance set at p < 0.05. Statistical analyses were conducted using SPSS software version 26.0 by IBM Corp., Armonk, NY.

The research adhered to ethical principles outlined in the Declaration of Helsinki (2000), WHO Guidelines for Ethical Committees for the Review of Biomedical Research (2000), and International Ethical Guidelines for Biomedical Research Involving Human Subjects (2002), in addition to the policies of the Research Advisory Committee (RAC) at King Faisal Specialist Hospital and Research Centre. The study was reviewed and approved by the Research Ethics Committee (REC) at King Faisal Specialist Hospital and Research Centre, assigned RAC number 2241149, on May 29, 2024.

The primary objective was to ascertain whether there is a significant correlation between elevated CRP levels (greater than 1 mg/L) and plantar fasciitis in patients with cardiovascular risk factors. The secondary objective was to compare the prevalence of diabetes, obesity, hypertension, and dyslipidemia between patients with plantar fasciitis and those with other causes of foot or ankle pain.

## Results

This is a non-interventional, cross-sectional quantitative study. We collected 400 patients who presented to the family medicine department complaining of foot or ankle pain and having at least one of the following cardiovascular risk factors: diabetes, hypertension, or dyslipidemia. We excluded 105 patients diagnosed with any disease that can cause elevated CRP, such as infections, acute or chronic inflammatory diseases such as rheumatoid arthritis, gout, Crohn’s disease, autoimmune disorders, cancers, or recent trauma. We also excluded any patient who is currently smoking cigarettes or shisha. The remaining 295 patients had a mean age of 58.26 (SD = 15.315); 53.5% were female, and 46.5% were males. 80.5% of all the patients were overweight or obese. 49.5% had diabetes type 2, 47.5% had hypertension, and 46% had dyslipidemia (Table 1).

**Table 1:** Demographic and clinical characteristics of the study population

Category	Details
Total number of patients assessed	400
Patients included in the final analysis	295
Mean age (years)	58.26
Standard deviation of age	15.315
Percentage female	53.5%
Percentage male	46.5%
Percentage overweight or obese	80.5%
Percentage with diabetes type 2	49.5%
Percentage with hypertension	47.5%
Percentage with dyslipidemia	46%

We examined the causes of foot pain in the remaining 295 patients. Almost half had foot pain secondary to plantar fasciitis (Table 2).

**Table 2:** Reasons for foot pain identified in the study participants.

Cause of the foot pain	Frequency	Percentage
Flat foot	16	5.4%
Old fracture/trauma	3	1.0%
Hallux valgus	6	2.0%
Osteoarthritis	85	28.8%
Neuropathic pain, such as sciatica or peripheral neuropathy	36	12.2%
Foot corns	8	2.7%
Plantar fasciitis	141	47.8%
Total	295	100%

In our study, there was no statistically significant association between gender (female or male) and the occurrence of plantar fasciitis. The Pearson chi-square value is 0.383, with a degree of freedom of 1 and a p-value of 0.536.

However, a statistically significant association exists between high BMI categories and plantar fasciitis. This is indicated by the Pearson chi-square value of 12.627 with a p-value of 0.027 and the likelihood ratio of 14.141 with a p-value of 0.015. We also found a statistically significant association between high CRP levels and the presence of diabetes (p-value = 0.0149), hypertension (p-value = 0.0243), and dyslipidemia (p-value = 0.0423). Similar results from other studies support these results [1-3].

We divided the patients into two groups according to the cause of the foot pain. In the first group, 141 (47.8%) patients had foot pain due to plantar fasciitis, and in the second group, 154 (52.2%) patients had foot pain due to any other cause.

There was no statistical difference between the two groups in the distribution of obesity (p-value = 0.074), hypertension (p-value = 0.165), dyslipidemia (p-value = 0.180), or diabetes (p-value = 0.3), chronic kidney disease (p-value = 1.000), or the distribution of vitamin D insufficiency or deficiency (p-value = 0.712).

To analyze the association between plantar fasciitis and high CRP levels, we categorized CRP levels into a binary variable (high for values greater than 1 mg/L and non-high risk for values less than 1 mg/L). Then, we conducted a chi-square test of independence to see if there was a statistically significant association between having plantar fasciitis and having a high CRP level. We found a statistically significant association (p-value = 0.035) between having plantar fasciitis and having high CRP (>1).

## Discussion

This study investigates the potential correlation between chronic plantar fasciitis and cardiovascular disease risk, as indicated by elevated CRP levels. In our cross-sectional study of 295 individuals presenting with foot or ankle pain, we observed a statistically significant association between plantar fasciitis and elevated CRP levels, suggesting systemic inflammation.

The prevalence of plantar fasciitis in our study population was notably high, affecting nearly half of the participants. Our findings align with the current literature that posits biomechanical imbalances and obesity as contributors to the development of plantar fasciitis [14,15]. In our cohort, a significant association between high BMI and plantar fasciitis was noted, underscoring the impact of obesity on musculoskeletal strain and inflammation.

Interestingly, while our study did not reveal a significant association between plantar fasciitis and other cardiovascular risk factors such as hypertension, dyslipidemia, or diabetes, we did find a significant association between elevated CRP levels and these conditions. This reflects the established understanding that systemic inflammation is a common thread linking various metabolic and cardiovascular disorders [6-9]. The elevated CRP levels in individuals with plantar fasciitis suggest that chronic heel pain may not merely be a localized issue but could also reflect broader systemic inflammation, which is a recognized risk factor for atherosclerosis and cardiovascular events.

In our study, plantar fasciitis occurred equally among all genders, indicating that gender differences do not influence the condition. This stands in contrast to certain other studies, where the frequency of occurrence is known to be higher among females [13].

Our study's novel finding is the association between plantar fasciitis and elevated CRP levels, which has yet to be conclusively established in the existing literature. The heightened CRP levels in individuals with plantar fasciitis, even when controlled for other cardiovascular risk factors, hint at the possibility that plantar fasciitis itself may be a marker of systemic inflammation and, therefore, a potential risk factor for cardiovascular disease. This observation warrants further investigation into the pathophysiological mechanisms linking localized plantar fascia inflammation with systemic inflammatory responses.

Moreover, our research raises the question of whether treating plantar fasciitis and reducing local inflammation could benefit systemic inflammation and reduce cardiovascular risk. It also opens the avenue for considering CRP levels as part of the assessment in patients with plantar fasciitis, particularly those with concurrent metabolic conditions.

These findings have significant implications for clinical practice. For patients presenting with chronic heel pain and concomitant cardiovascular risk factors, clinicians should consider the potential systemic implications of plantar fasciitis. Further research is needed to determine if targeted treatment strategies for plantar fasciitis could influence the progression of systemic inflammation and thereby modify cardiovascular risk profiles.

There are limitations to our study that warrant mention. First, the cross-sectional nature of the research design prevents us from establishing causality. Second, excluding patients with any disease known to cause elevated CRP levels could have resulted in selection bias. Additionally, the reliance on patient self-report for the diagnosis of plantar fasciitis without confirmatory imaging may affect the accuracy of the diagnosis. Future longitudinal studies are necessary to confirm these findings and to explore the causal relationship between plantar fasciitis, systemic inflammation, and cardiovascular disease.

This study opens several pathways for future research. Longitudinal studies investigating the temporal relationship between the onset of plantar fasciitis, changes in CRP levels, and the development of cardiovascular events would be particularly interesting. Also, interventional studies assessing the impact of treating plantar fasciitis on CRP levels and cardiovascular risk factors would provide insight into potential benefits beyond symptom relief.

## Conclusions

The study indicates that patients with diabetes, hypertension, or dyslipidemia who also have plantar fasciitis are more likely to have systemic inflammation, as evidenced by a significant correlation between plantar fasciitis and elevated CRP levels. This condition of heightened CRP levels, more prevalent in those with plantar fasciitis, may suggest a heightened risk of cardiac issues. Hence, in those presenting with cardiovascular risk factors like diabetes, high blood pressure, or dyslipidemia, the presence of plantar fasciitis might act as an indicator of systemic inflammation that could potentially increase the risk of developing ischemic heart disease.
